# An Umbrella Review of Quality of Life Among the General Population During the COVID-19 Pandemic

**DOI:** 10.3390/jcm14238348

**Published:** 2025-11-24

**Authors:** Paul Watts, Alexandra Deac, Gopalakrishnan Netuveli

**Affiliations:** 1 School of Health, Sport and Bioscience, University of East London, Water Lane, London E15 4LZ, UK; p.n.watts@uel.ac.uk; 2 Institute for Connected Communities, University of East London, Water Lane, London E15 4LZ, UK; a.deac@uel.ac.uk

**Keywords:** COVID-19, pandemic, quality of life, systematic review, umbrella review, wellbeing

## Abstract

**Background/Objectives**: The COVID-19 pandemic has had widespread effects on quality of life (QoL). This umbrella review aimed to synthesise review-level evidence on the impact of the COVID-19 pandemic on QoL across the general population, including children, adolescents, adults, and older people. **Methods**: A systematic search of databases (MEDLINE (PubMed); EBSCOHost; Scopus; Google Scholar) and review repositories was conducted to identify systematic reviews, meta-analyses, and scoping reviews published between 2019 and 2025. Eligible reviews (2019–2025, English, peer-reviewed) included narrative, systematic and meta-analytic syntheses of quantitative, qualitative or mixed-methods evidence examining the impact of the COVID-19 pandemic on quality of life in the general population, using validated QoL measures. The review followed Joanna Briggs Institute methodology for umbrella reviews, with data synthesised narratively by QoL domain and population group. **Results**: Nine reviews met the inclusion criteria, encompassing 272 primary studies. Most reported declines in QoL across psychological, physical, and social domains. Children and adolescents experienced reductions in emotional and social functioning. Working-age adults reported psychological strain linked to economic and health-related stressors, while older adults were vulnerable to isolation and reduced social QoL. Environmental and structural factors also influenced QoL. Sociodemographic disparities were observed, with lower QoL reported among women, younger individuals, and those with lower socioeconomic status. **Conclusions**: The COVID-19 pandemic was associated with substantial declines in QoL across population groups, shaped by psychological, social, and structural factors. Findings highlight the importance of addressing social inequalities and enhancing resilience in future public health crises.

## 1. Introduction

The COVID-19 pandemic outbreak in 2019 has had profound and multifaceted effects on many aspects of human life [1]. Since the World Health Organization (WHO) declared COVID-19 a global emergency in 2020 [2], the pandemic has persisted for several years, leaving lasting impacts on physical health, mental health [3,4], and quality of life [5]. The concept of quality of life (QoL) is defined by WHO as “an individual’s perception of their position in life in the context of the culture and value systems in which they live and in relation to their goals, expectations, standards and concerns” [6]. QoL can be understood as comprising four key domains: psychological, physical, social, and environmental [7,8]. This multidimensional concept captures the interplay between personal goals, societal expectations, and the capacity to achieve a sense of well-being. QoL is an essential metric to capture the comprehensive impact of the COVID-19 pandemic on the general population beyond mortality and infection rates [9]. In this umbrella review, we adopt this WHO definition of QoL to map the current evidence on the impact of the COVID-19 pandemic on QoL as subjective well-being and evaluations of satisfaction within multifaceted life domains among the general population [8].

There is evidence to suggest that disruptions to social structures, economic systems and daily routines have significantly impacted QoL across all age groups [10,11,12]. There is also evidence of differential impacts across groups, indicating that some more quickly adapted to the conditions of the pandemic, improving their health and life satisfaction, while others experienced elevated stress levels and poorer health outcomes [13,14]. For example, a recent systematic review by Duan et al. [15] provided a synthesis of the impact of the COVID-19 virus on the health and mental health of children and adolescents. The review findings suggest that in addition to physical health impacts such as respiratory and hematologic symptoms, the impacts of the pandemic included increased mental health impacts including depression and anxiety. Evidence from primary studies on the impact of COVID-19 on QoL among older adults appears mixed. Several primary studies report initial declines in mental health among older adults [16], though others highlight resilient responses [17]. Overall, older adults are commonly identified as an at-risk group [18], with studies reporting reduced QoL due to increased isolation and poorer physical and mental health outcomes [19]. Systematic reviews and meta-analyses indicate that mental health among older adults worsened during the early stages of the pandemic (2020 to 2021), followed by signs of recovery in later stages from 2022 onwards [20,21,22].

Evidence from some systematic reviews suggests that QoL among the general population may have worsened during the COVID-19 pandemic, although findings are nuanced and may vary by population group, life domain, and contextual factors [23]. Some studies have described the pandemic in terms of collective trauma [24] while others indicate resilient and recovering trends. A recent systematic review and meta-analysis assessed the impact of the COVID-19 pandemic on the QoL among the general population, with findings suggesting that specific domains such as sociodemographic factors, financial instability and chronic diseases severely impacted the perceived QoL among the general population [25]. There is evidence to suggest that lockdowns, fear of infection and social isolation are associated with poorer QoL among the general population during the pandemic [26]. A recent study conducted a cross-sectional analysis of 13 high and low- and middle-income countries, comparing the QoL among the general population during the pandemic [27]. The results indicate an overall reduction in perceived QoL, with women and younger adults (under 35) reporting poorer mental health outcomes.

Despite broad evidence on the impact of the pandemic on the QoL, most reviews focus on specific subgroups [15,28], making it challenging to understand the links between QoL and the impact of the pandemic among the general population. Additionally, the large and overlapping body of systematic reviews and meta-analyses in this area presents challenges for interpretation and synthesis. This umbrella review aims to comprehensively map the existing reviews on the impact of the COVID-19 pandemic on QoL among the general population (globally) at different life stages: children; adolescents; working-age adults; older adults. By compiling existing evidence from multiple reviews, this umbrella review will provide a holistic, high-level understanding of the impact of the pandemic on the general population. The findings will identify common patterns and gaps in research, informing policymakers, healthcare providers and other stakeholders in designing interventions that help to mitigate the negative impacts of future public health crises.

## 2. Materials and Methods

### 2.1. Review Question

The objective of this umbrella review is to synthesise and evaluate the evidence from systematic reviews on the impact of the COVID-19 pandemic on QoL. The review aims to identify key factors associated with QoL during the pandemic and provide a comprehensive understanding of the findings reported in existing reviews. The specific review question is as follows: What are the reported impacts of the COVID-19 pandemic on quality of life across different life course stages within the general population?

### 2.2. Study Design

This umbrella review was conducted using the Joanna Briggs Institute (JBI) methodology for umbrella reviews [29] to synthesise evidence from systematic reviews and meta-analyses examining the impact of the COVID-19 pandemic on QoL across the life course. This design allows for the integration of findings from multiple reviews to provide a comprehensive understanding of the factors influencing QoL during the pandemic. There is currently no PRISMA checklist extension specific to umbrella reviews. We therefore applied PRISMA 2020 guidance where appropriate and completed the PRISMA 2020 Checklist and PRISMA 2020 for Abstracts Checklist, which are provided as Supplementary Materials (Tables S1 and S2). We additionally completed the PRIOR Checklist for Overviews of Reviews of Healthcare Interventions (Table S3). Although the PRIOR checklist is intended for reviews of healthcare interventions rather than for evidence syntheses of non-interventional studies, it was included to enhance transparency and completeness of reporting.

### 2.3. Inclusion Criteria

#### 2.3.1. Types of Studies

This umbrella review included reviews of quantitative, qualitative, and mixed-methods designs that assessed the effects of the COVID-19 pandemic on QoL during and after the pandemic. Eligible reviews included narrative reviews, systematic reviews and meta-analyses that adhered to established review methodologies [30]. Reviews were required to be published in peer-reviewed journals between 2019 and 2025, in the English language. Non-review articles (e.g., commentaries, perspectives, book chapters, letters, conference abstracts, or grey literature) were excluded.

#### 2.3.2. Types of Participants

The included reviews examined the general population, defined as all inhabitants of a geographic area at a specific point in time, irrespective of specific sociodemographic characteristics [31]. Reviews considering specific subgroups, such as healthcare workers or populations with pre-existing mental health diagnoses or chronic comorbidities, were excluded. Consistent with our general population focus, we also excluded reviews that only included studies focusing on COVID-19 ‘patients’, ‘survivors’ or individuals with a confirmed COVID-19 diagnosis.

#### 2.3.3. Phenomena of Interest

The phenomenon of interest was the impact of the COVID-19 pandemic on QoL within the general population. The COVID-19 pandemic was defined not only as a global outbreak of coronavirus disease caused by SARS-CoV-2 but also as a broader social phenomenon, encompassing societal responses and contexts during the pandemic period. Reviews were included if they explicitly assessed QoL in the context of the pandemic, moving beyond epidemiological impacts to consider social, cultural, and economic factors.

#### 2.3.4. Outcomes

The primary outcome of interest was QoL, as defined by the World Health Organization: “an individual’s perception of their position in life in the context of the culture and value systems in which they live and in relation to their goals, expectations, standards, and concerns” [6]. Reviews were included if they used validated QoL measures (e.g., EQ-5D (EuroQol 5 Dimensions), WHOQOL-BREF (World Health Organization Quality of Life-BREF), and SF-36 (Short Form Health Survey-36 items)) or qualitative assessments aligned with this definition. Reviews focusing on interventions to improve QoL or those not explicitly assessing the impact of the COVID-19 pandemic on QoL were excluded.

#### 2.3.5. Context

The context for this umbrella review was the global COVID-19 pandemic and its impacts on QoL. Reviews specific to the general population during this period were included, while those focusing exclusively on subpopulations (e.g., healthcare workers) or contexts unrelated to the pandemic were excluded.

### 2.4. Search Strategy

A comprehensive search strategy was undertaken to identify relevant systematic reviews and meta-analyses addressing the effects of the COVID-19 pandemic on QoL. The search aimed to retrieve all eligible reviews, published between 2019 and 2025, in the English language. Although the COVID-19 pandemic began in December 2019, and it is therefore unlikely that any reviews were published that year, we included 2019 in the search range to ensure that any early or misclassified publications could be identified. Initial searches were conducted to identify keywords and index terms relevant to the review question. Text words contained in the titles and abstracts of identified articles, as well as index terms used to describe the reviews, were analysed.

Searches were performed across the following electronic databases: MEDLINE (PubMed); EBSCOHost (including Academic Search Ultimate, CINAHL Complete, APA PsycArticles, and APA PsycInfo); Scopus; Google Scholar. The following systematic review repositories and registers were also searched: Cochrane Database of Systematic Reviews; PROSPERO; Epistemonikos. Search filters were designed to identify reviews and syntheses of evidence, using key terms such as “systematic review,” “meta-analysis,” or “synthesis” in combination with terms for QoL (e.g., “Quality of Life,” “QoL”) and the COVID-19 pandemic (e.g., “COVID-19,” “SARS-CoV-2,” “Coronavirus”). An example of a database-specific search string used in PubMed is: ((“Quality of Life” [Title] OR “quality of life” [Title] OR “QoL” [Title]) AND (“COVID-19” [Title] OR “COVI*” [Title] OR “SARS-CoV-2” [Title] OR “Coronavirus” [Title] OR “2019-nCoV” [Title] OR “Coronavirus Disease 2019” [Title]) AND (“review” [Title] OR “Synthesis” [Title] OR “meta-analysis” [Title])). Reference lists of all included reviews were hand-searched for additional relevant articles.

The final search was conducted on 8 July 2025, ensuring that all relevant literature published up to that date was considered. Search filters and inclusion criteria (e.g., language and date limits) were consistently applied across all databases and sources.

### 2.5. Study Selection

**Figure 1:**
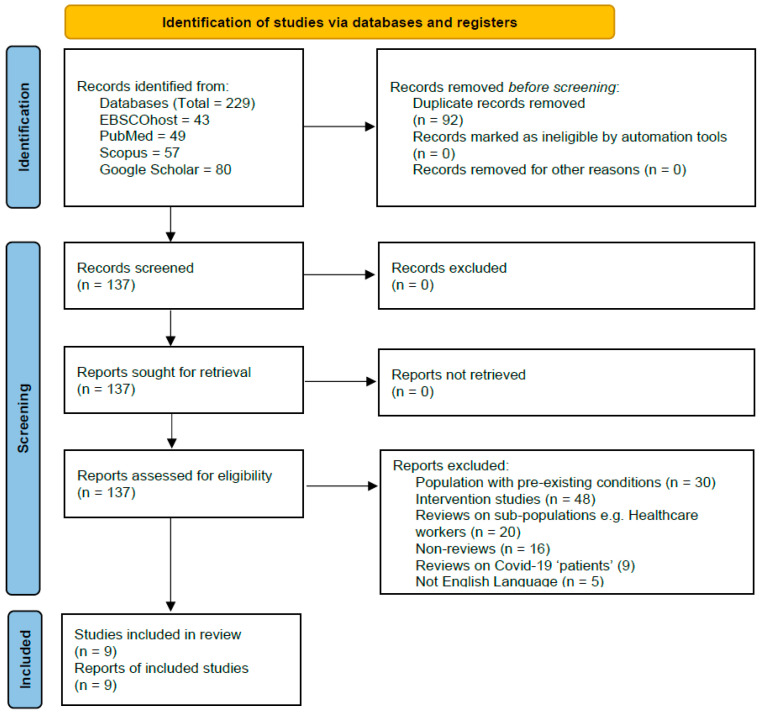
PRISMA flow diagram showing number of records identified, included, and excluded at each stage, and the reasons for exclusions.

Two reviewers (PW and AD) independently screened the titles and abstracts of all records identified through the search strategy. Articles were included for full-text review if they met the inclusion criteria or if there was insufficient information to make a decision at this stage. The full text of all potentially eligible articles was then retrieved and reviewed independently by the two reviewers. Reviews were included if they met all inclusion criteria and did not meet any of the exclusion criteria. Any disagreements between reviewers during either stage of the selection process were resolved through discussion and consensus. If consensus could not be reached, a third reviewer (GN) was consulted to make a final decision. The number of articles included and excluded at each stage of the selection process was recorded, and reasons for exclusion at the full-text stage were documented. The study selection process is presented in a PRISMA flow diagram (Figure 1).

### 2.6. Assessment of Methodological Quality

Two reviewers (PW and AD) independently evaluated the methodological quality of the included studies using the JBI Critical Appraisal Checklist for Systematic Reviews and Research Syntheses [32]. Both reviewers are experienced researchers with expertise in public health, QoL research and evidence synthesis. This tool assesses the extent to which each review has addressed potential bias in its design, implementation, and analysis. The checklist consists of 11 criteria, each rated as “yes,” “no,” “unclear,” or “not applicable.” Any disagreements between the reviewers were resolved through discussion and consensus. If consensus could not be reached, a third reviewer (GN) was consulted to make a final rating.

To calculate quality scores, we adopted a method used in previous umbrella reviews [33]. For each study, the total score was determined by dividing the number of criteria rated “yes” by the maximum number of applicable criteria, yielding a percentage score. Based on this score, studies were categorised as low quality (0–33% of criteria met), medium quality (34–66% of criteria met), or high quality (67% or more of criteria met). The scoring system assigned 1 point for “yes” (criterion fully met), 0 points for “no” (criterion not met), and 0 points for “unclear” (insufficient information). Criteria marked as “not applicable” were excluded from the calculation, with the denominator adjusted accordingly.

### 2.7. Data Extraction

Data were extracted independently by two reviewers (PW and AD) using a pre-designed Excel data extraction sheet tailored for this umbrella review. The tool captured key information, including review characteristics (e.g., title, authors, publication year, journal, review type, objectives, and population focus), methods (e.g., inclusion and exclusion criteria, databases searched, and search strategies), and study characteristics (e.g., number of included studies, study designs, participant demographics, and quality appraisal methods). Specific details related to QoL, such as measurement tools, reported outcomes, and factors influencing QoL (e.g., social, mental, and physical health determinants), were also extracted.

### 2.8. Data Synthesis

A narrative synthesis approach was employed to integrate findings across the included reviews, following methodological guidance for umbrella reviews [30]. The synthesis was structured around key domains of QoL aligned with the WHO definition, and organised by population group, exposure context, and life domain. The process involved data reduction through thematic grouping (e.g., psychological, physical, social, environmental), comparison of patterns within and across reviews, and conclusion drawing based on consistency and strength of findings. Themes were derived iteratively, with subgroup analysis (e.g., by age, gender, or region) used to identify differential impacts. Primary studies cited in the results are limited to instances where specific findings or details were not included in the summaries of the included reviews.

### 2.9. Study Overlap

To assess the degree of overlap among primary studies included in the systematic reviews, we constructed an overlap matrix listing each primary study against the reviews in which it appeared. Overlap was quantified using the Corrected Covered Area (CCA) as described by Pieper et al. [34]. The overlap matrix was compiled in Microsoft Excel, and all calculations were conducted using standard spreadsheet formulae. Two reviews [7,35] did not fully report their included primary studies, precluding their inclusion in the overlap analysis.

## 3. Results

### 3.1. Overview of Included Reviews

A total of nine reviews met the inclusion criteria for this umbrella review, encompassing 272 primary studies.

#### 3.1.1. Types of Studies

Key study characteristics of each review are summarised in Table 1 and methodological details are summarised in Table 2. The included reviews were published between 2020 and 2024, with most published during the early phases of the pandemic (2020–2022) and a smaller number offering more reflective or longitudinal evidence in later years. Seven reviews adopted a systematic review methodology, with two of these incorporating meta-analyses [5,25]. One used a scoping review framework to map emerging evidence across a heterogenous literature base [14]. One review conducted a structured synthesis of literature on QoL and urban planning, without using a structured systematic review process [7].

The number of studies included in each review ranged from 6 [14] to approximately 100 [7], reflecting differences in scope and inclusion criteria. Most primary studies included in the reviews were cross-sectional in design, particularly those from the early stages of the pandemic. A smaller number of longitudinal studies were reported, particularly in reviews focused on children and adolescents [36,37], allowing for assessment of changes in QoL over time.

#### 3.1.2. Types of Participants

Four reviews examined QoL in the general population without age restriction [5,7,25,35]. Three reviews focused on children and adolescents [36,37,38] and one examined the QoL of older adults [14]. Another review focused on populations from countries severely affected in the early phases of the pandemic, such as Italy, Saudi Arabia, China, and Vietnam [39]. Across reviews, demographic reporting was inconsistent, but where available, studies spanned the life course and often included gender, age, and, less frequently, socioeconomic status disaggregation.

#### 3.1.3. Phenomena of Interest

All reviews included focused on the effects of the COVID-19 pandemic on QoL. The scope was limited to the pandemic period, with no reviews restricted to post-pandemic impacts. Some reviews focused primarily on the early pandemic [39], while others included data through 2021 and 2022 [25,37], capturing more sustained or evolving impacts.

#### 3.1.4. Outcomes

The included reviews examined a range of QoL outcomes. Most commonly, these were overall health-related QoL. Several reviews applied a multidimensional framework consistent with the World Health Organization’s definition of QoL, incorporating physical, emotional, social, and environmental domains [7]. Others focused more narrowly on specific aspects of emotional or social functioning, particularly in younger populations [7].

A variety of validated measurement tools were used across the reviews. The WHOQOL-BREF and SF-36 were the most commonly reported instruments in general population studies [5,25]. The European Quality of Life Survey (EQLS) was also employed in some studies [25]. In studies involving children and adolescents, age-appropriate tools such as the Pediatric Quality of Life Inventory (PedsQL) and KIDSCREEN were used [36]. A small number of reviews did not report specific QoL tools, instead referring to broader constructs of QoL without standardised measurement being defined [7,14,35].

#### 3.1.5. Context

Geographically, most reviews adopted a global perspective, with studies drawn from a wide mix of high-income and low- and middle-income countries. Several reviews offered regional analyses, for example, Pashazadeh Kan et al. [5] reported QoL scores by WHO region. While some reviews included only a limited number of countries [36,39], the majority sought to synthesise internationally relevant findings.

**Table 1:** Summary of the main study characteristics of included reviews.

First Author	Year	Review Type	Objectives/Aim of the Review	Population Focus	No. of Studies Included
El Keshky [35]	2020	Systematic review	To evaluate the impact of COVID-19 on the psychology of sustainability (quality of life).	General population	61
Kasar [14]	2021	Scoping review	To evaluate the quality of life of elderly individuals during the COVID-19 pandemic.	Older Adults	7
Melo-Oliveira [39]	2020	Systematic review	To summarize the effects of COVID-19 on the quality of life (QoL) of studied populations.	General Population	8
Mouratidis [7]	2021	Narrative Review	To understand how COVID-19 reshaped the relationship between cities and quality of life.	General population	Approx. 100
Nae Ahn [36]	2022	Systematic review	To investigate changes in child and adolescent HRQoL before and during the pandemic	Children and Adolescents	8
Nobari [38]	2021	Systematic review	To assess the impact of the COVID-19 pandemic on HRQoL of children and adolescents.	Children and Adolescents	6
Nshimirimana [25]	2023	Systematic review and meta-analysis	To assess the impact of COVID-19 and associated factors on Health-Related Quality of Life.	General Population	25 for qualitative synthesis; 17 for quantitative analysis
Orban [37]	2024	Systematic review	To examine longitudinal studies to understand the long-term impacts of the pandemic on children and adolescents.	Children and Adolescents	24
Pashazadeh Kan [5]	2023	Systematic review and meta-analysis	To provide evidence-based information on the impact of the COVID-19 pandemic on quality of life (QOL).	General Population	33

**Table 2:** Summary of the methodological details of included reviews.

First Author	Date Range of Included Studies	Geography of Included Studies	Study Designs in Included Studies	Total Sample Size (All Studies)	Quality Appraisal/Risk of Bias Assessment Used	QoL Measurement Tools Used in Included Studies
El Keshky [35]	2010 to 2020	Global	Not reported	Not reported	Not reported	Not reported
Kasar [14]	December 2019 to March 2021	Global	Descriptive; cross-sectional; quasi-experimental study; pre-post pilot; editorial; correspondence	Not reported	Not reported	Not reported
Melo-Oliveira [39]	Onset of COVID-19 to May 2020	Italy; Saudi Arabia; China; Vietnam	Not reported	7051	PEDro scale	CVID-QoL; GHQ-12; IES-15; SRQ; PSQI; HLSSF12; PHQ-9; SF-36
Mouratidis [7]	Published before September 2021	Global	Not reported	Not reported	Not reported	Not reported
Nae Ahn [36]	2020 to March 2022	The United States, Norway, Spain, Southern Germany, France	Cross-sectional	20,509	Evidence Project Risk of Bias Tool	PedsQL; KIDSCREEN-10; KIDSCREEN-27; KINDL-R; CPQ11–14
Nobari [38]	2020 to 2021	Netherlands, Croatia, Brazil, Germany, Spain	Cross-sectional	3177	Evidence Project Risk of Bias Tool	PedsQL; SF-36; KIDSCREEN-10
Nshimirimana [25]	From the onset of COVID-19 to 2023	Global	Cross-sectional; mixed methods	22,967	Modified Newcastle-Ottawa Scale	WHOQoL-BREF; EQ-5D; SF-12/SF-8/SF-36; EQLS; GHQ-12
Orban [37]	2021 to 2022	Global	Longitudinal	Not reported	Effective Public Health Practice Project (EPHPP) checklist	KIDSCREEN
Pashazadeh Kan [5]	2019-May 2021	Global	Descriptive, prospective, cross-sectional, case-series and cohort studies	23,058	Newcastle-Ottawa Scale	SF-36 OR WHOQOL-BREF

PHQ-9 = Patient Health Questionnaire-9; PSQI = Pittsburgh Sleep Quality Index; IES-15 = Impact of Event Scale-15; SRQ = Self-Reporting Questionnaire; SF-12 = Short Form Health Survey-12; SF-36 = Short Form Health Survey-36; SF-8 = Short Form Health Survey-8; EQ-5D = EuroQol Five-Dimension Scale; WHOQOL-BREF = World Health Organization Quality of Life—Brief; KIDSCREEN = KIDSCREEN-10 Health-Related Quality of Life Questionnaire; PedsQL = Pediatric Quality of Life Inventory; GHQ-12 = General Health Questionnaire-12; EQLS = European Quality of Life Survey; HLSSF12 = Health and Lifestyle Survey Form-12; PEDro = Physiotherapy Evidence Database Scale; EPHPP = Effective Public Health Practice Project Quality Assessment Tool.

### 3.2. Quality Appraisal

The methodological quality of the nine included reviews was assessed using the JBI Critical Appraisal Checklist for Systematic Reviews and Research Syntheses. Overall, five reviews were rated as high quality, three as medium, and one as low quality. Most reviews clearly stated their review question, applied appropriate inclusion criteria, and used robust search strategies and sources. However, critical appraisal methods varied considerably. Only five reviews reported conducting critical appraisal using appropriate criteria, and only five confirmed independent appraisals by multiple reviewers. Several reviews lacked clarity on methods used to minimise errors in data extraction or to combine study findings, which limited transparency in synthesis. In general, reviews scored lower on items relating to critical appraisal processes and synthesis methods than on those related to question clarity and search strategy. Table 3 provides a full summary of quality ratings. Despite some methodological limitations, the majority of reviews included in this umbrella review were of moderate to high quality, supporting confidence in the overall findings.

### 3.3. Study Overlap

The overlap analysis indicated minimal duplication of primary studies across the included reviews. After excluding two reviews that did not fully report their included studies, the Corrected Covered Area (CCA) was 0.63%, representing slight overlap according to established thresholds [34].

### 3.4. Psychological Dimensions of QoL

#### 3.4.1. Psychological Dimensions of QoL—Children and Adolescents

Across three reviews, the psychological dimensions of QoL in children and adolescents were found to have declined during the COVID-19 pandemic, with emotional well-being and psychological functioning identified as particularly affected domains [36,37,38]. Emotional distress linked to school closures, social isolation, and family pressures was consistently associated with lower QoL scores, especially on instruments such as the PedsQL and KIDSCREEN.

In a systematic review of 8 studies, Ahn [36] reported frequent impairments in emotional functioning, including increased irritability, nervousness, and unhappiness, all of which were directly associated with reduced health-related quality of life (HRQoL). Similar patterns were observed by Nobari et al. [38], who reviewed six studies and found psychological functioning to be the most affected QoL domain. Significant declines were noted in emotional well-being, vitality, and coping, with one study showing that the proportion of children reporting low emotional QoL more than doubled during lockdown [40]. Although two studies in that review did not find statistically significant differences [41,42], the weight of evidence pointed to a clear overall deterioration.

Reviewing evidence from longitudinal studies, the review by Orban et al. [37] highlighted that disruptions to emotional QoL were often sustained or intensified as the pandemic progressed. Children’s difficulties with emotional regulation and coping under prolonged restrictions were cited as key contributors to declining perceptions of their general QoL. The extent of this decline appeared to be associated with factors such as access to social support and the strictness of lockdown measures.

#### 3.4.2. Psychological Dimensions of QoL—Working-Age Adults

The psychological dimension of QoL among working-age adults was an area of focus across two reviews, both of which reported reductions in mental well-being and emotional functioning during the COVID-19 pandemic. In their meta-analysis of 25 studies, Nshimirimana et al. [25] reported a pooled mental component summary (MCS) score of 51.6 (95% CI: 47.3–55.9), suggesting diminished psychological QoL in the general adult population.

#### 3.4.3. Psychological Dimensions of QoL—Older Adults

Evidence from the scoping review by Kasar and Karaman [14] indicates that older adults experienced declines in psychological well-being that contributed to reduced QoL during the COVID-19 pandemic. Across the seven studies included, social isolation and loneliness emerged as central drivers of diminished emotional functioning, particularly among those living alone or with limited access to digital communication.

Several studies cited in the review reported a direct link between increased loneliness and lower scores on the mental health domains of QoL measures. In Austria and Switzerland, cross-sectional studies found that older adults experienced heightened loneliness during lockdowns, with corresponding reductions in emotional well-being and life satisfaction [43,44]. These effects were most pronounced among individuals with minimal social support or digital connectivity.

Other studies noted positive associations between emotional functioning and optimism, social support, or the availability of social interaction. One study found that older adults reported higher psychological functioning than younger adults during the same period [45], though this was an outlier within a broader pattern of decline.

### 3.5. Physical Dimensions of QoL

#### 3.5.1. Physical Dimensions of QoL—Children and Adolescents

In the review by Ahn [36], multiple studies noted a decline in children’s physical activity levels during lockdown periods, which corresponded with lower physical functioning scores on the PedsQL. Children reported decreased energy levels, more frequent fatigue, and reduced capacity to engage in play or physical tasks, all of which contributed to lower overall HRQoL. These effects were consistent across studies using both self- and parent-reported measures.

Nobari et al. [38] also found that pandemic-related restrictions led to marked reductions in physical health-related QoL, with contributing factors including decreased exercise opportunities and elevated screen time. One included study using the SF-36 reported statistically significant reductions in all physical domains of QoL, including functional capacity, general health, and vitality, in both children and adolescents [46]. Another study using the KIDSCREEN-10 reported that pre-pandemic physical activity levels positively predicted QoL during the pandemic [47], suggesting that more active children may have been somewhat buffered from the negative impact.

#### 3.5.2. Physical Dimensions of QoL—Working-Age Adults

QoL in working-age adults during the pandemic was also shaped by changes in physical health and functional capacity, as evidenced in two reviews that identified healthcare disruption and reduced physical activity as key contributors to declines in physical QoL [25,35]. Nshimirimana et al. [25], reported a pooled physical component summary (PCS) score of 60.1 (95% CI: 53.2–67.0) across 25 studies, indicating moderate impairment in physical QoL. These scores were lower in populations with reduced access to healthcare or chronic illness, and in regions more heavily impacted by pandemic-related restrictions. The review also highlighted that delays in routine medical care, disruptions to physical rehabilitation, and restricted access to health services were common themes in studies reporting poor physical QoL outcomes.

Similarly, El Keshky et al. [35] noted that health service access was severely constrained during lockdowns, particularly in lower-income countries with fragile health infrastructures. This contributed to reduced ability to manage existing health conditions, which in turn impaired daily functioning and physical well-being. Several studies cited in the review linked pandemic-related limitations, such as reduced mobility, decreased exercise, and avoidance of medical facilities, with lower perceptions of physical health and QoL.

#### 3.5.3. Physical Dimensions of QoL—Older Adults

Among older adults, the physical dimension of QoL was impacted during the pandemic, primarily due to reduced mobility, disruption of routine health services, and increased frailty. Kasar and Karaman [14] reported that lockdown measures led to substantial decreases in physical activity and increased sedentary behaviour, contributing to functional decline in older populations. The loss of structured routines and support systems exacerbated pre-existing health conditions and heightened risks of falls, malnutrition, and medication mismanagement. Similarly, Nshimirimana et al. [25] identified diminished scores in HRQoL among older adults. Included studies using the EQ-5D reported that in the general population, the likelihood of experiencing pain, discomfort, and symptoms of anxiety or depression increased with age.

### 3.6. Social Relationships & Isolation

#### 3.6.1. Social Relationships & Isolation—Children and Adolescents

Ahn’s systematic review [36] of 20 studies identified consistent reductions in social functioning and peer relationships during the pandemic, as reflected in validated QoL instruments such as KIDSCREEN and the PedsQL. Included studies found mixed evidence of the impacts of school closures and distancing measures associated with lower scores on social support and peer interaction subscales, particularly among adolescents [48,49]. One study included in Ahn’s review reported a significant decrease in social support scores during the pandemic using the KIDSCREEN-10, highlighting the erosion of peer-connectedness in this age group [50].

Orban et al. [37] synthesised longitudinal evidence showing that these declines in the social dimension of QoL often persisted beyond the initial lockdowns. Among the four HRQoL studies included in their review, social functioning was assessed using the KIDSCREEN across multiple time points. For instance, the German COPSY study found that peer and social support scores remained lower than pre-pandemic levels at all measured intervals, including after the easing of restrictions [40]. Similarly, the Norwegian COVID-19 Young study found that while some aspects of HRQoL improved over time, peer and social support increased only modestly and remained below expected norms [51]. These patterns suggest a slower recovery in social functioning compared to other QoL domains, particularly for adolescents whose social development is more reliant on sustained peer interaction.

#### 3.6.2. Social Relationships & Isolation—Working-Age Adults

Mouratidis [7] identified digital communication technologies as a factor influencing QoL during the COVID-19 pandemic, particularly in the context of social connectedness. This narrative synthesis of early pandemic research from urban settings, noted that the shift to online modes of interaction, such as teleworking, digital socialising, and virtual leisure, helped maintain certain aspects of daily life and social contact, which in turn supported self-reported QoL. The review emphasised that the presence of robust information and communication technology (ICT) infrastructure enabled continuity in social engagement despite physical distancing, particularly in cities. However, the review also highlighted that the capacity to benefit from digital substitution was not evenly distributed. Groups with limited access to digital tools—including older adults, people with low income, or residents of areas with inadequate connectivity—were less able to sustain social interaction during restrictions. This digital divide was linked to lower levels of reported social satisfaction and overall QoL in affected populations.

#### 3.6.3. Social Relationships & Isolation—Older Adults

The scoping review by Kasar and Karaman [14] consistently found that for older adults, reductions in social contact, particularly among those living alone or without access to digital communication tools, negatively influenced emotional well-being and social functioning—key components of QoL. Several studies identified increased loneliness as a prominent outcome of pandemic-related restrictions, with direct consequences for perceived QoL. In Austria, Stolz et al. [43] found that older adults living alone reported significantly higher levels of loneliness during lockdown, which were associated with declines in emotional well-being. Similarly, Macdonald and Hülür [44] reported that in Switzerland, older adults with reduced social interaction experienced lower levels of life satisfaction.

Furthermore, there was evidence that among older adults, especially those living alone, limited use of ICT was associated with persistent social isolation and reduced emotional well-being, both of which contributed to poorer QoL assessments [52]. The scoping review by Kasar & Karaman [14] also highlighted digital exclusion as a compounding factor. While some older adults were able to maintain social contact through technology, others faced barriers due to lack of access or familiarity with digital platforms.

### 3.7. Environmental & Structural Factors

#### 3.7.1. Environmental & Structural Factors—WHO Region Variability

Pashazadeh Kan et al. [5] conducted a systematic review and meta-analysis examining global and regional variations in QoL during the COVID-19 pandemic using data from 33 studies encompassing 23,058 participants. The review provided a comparative analysis based on WHO regions, revealing significant geographical disparities in reported QoL. The meta-analysis showed that average QoL scores during the pandemic differed markedly by region. The highest mean QoL score was observed in the Region of the Americas (AMRO) at 66.77 (95% CI: 60.55–73.00), followed by the Western Pacific Region (WPRO) at 64.79 (95% CI: 59.30–70.28). In contrast, the South-East Asia Region (SEARO) recorded the lowest average QoL score at 47.95 (95% CI: 47.67–48.23), a level classified by the authors as indicating poor QoL. Other regions such as the Eastern Mediterranean Region (EMRO: 52.03), African Region (AFRO: 53.27), and European Region (EURO: 58.51) fell within a moderate QoL range but still showed considerable heterogeneity in outcomes.

These findings were derived from studies using validated QoL instruments including the SF-36 and WHOQOL-BREF. The review highlighted that the observed regional disparities may reflect differences in health system capacity, the strictness and duration of lockdown measures, and underlying socioeconomic conditions. Additionally, subgroup analysis suggested that gender and age also interacted with regional context to influence QoL outcomes—men and younger populations tended to report higher scores across regions.

#### 3.7.2. Environmental & Structural Factors—Urban Infrastructure

The review by Mouratidis [7] examined how urban infrastructure shaped experiences of QoL during the COVID-19 pandemic. Drawing on international evidence, the review identified that access to green and blue space, housing quality, and local walkability contributed to sustaining perceived QoL under pandemic conditions, particularly in dense urban areas. Green and blue spaces were reported to support subjective QoL by offering residents safe settings for leisure and daily activity when movement was otherwise restricted. Studies included in the review found that such spaces helped maintain life satisfaction and environmental QoL during lockdowns. For example, Poortinga et al. [53] reported that public and private green space availability was positively associated with self-reported QoL in the UK. Similarly, Pouso et al. [54] found that regular contact with green and blue areas (e.g., parks, coasts) was linked to higher subjective QoL scores during periods of strict confinement.

Housing quality also emerged as a significant factor. The review noted that dwelling size, natural lighting, and access to private outdoor space contributed to perceived residential QoL. In one study, Amerio et al. [55] found that inadequate housing conditions such as overcrowding and a lack of private space were associated with poorer self-rated QoL and life satisfaction among residents under lockdown in Italy.

Walkability and proximity to essential services supported QoL by enabling safe and autonomous access to shops, healthcare, and public space [7]. The Mouratidis review highlighted that compact urban environments allowed residents to continue basic routines during restrictions, contributing to a sense of normalcy and maintaining aspects of day-to-day QoL. However, it also emphasised that these benefits were unequally distributed. Individuals in car-dependent, poorly connected neighbourhoods or with limited access to green space reported lower levels of QoL.

### 3.8. Variation in QoL by Sociodemographic Factors

#### 3.8.1. Age

In general population studies, older adults tended to report better QoL than younger individuals. Nshimirimana et al. [25] found that younger adults consistently reported lower scores on both physical and mental health domains of QoL, with older adults showing greater resilience, particularly in the mental component. Similarly, a study reviewed by Kasar and Karaman (2021) found that older adults rated their QoL higher than younger adults during the pandemic, despite being at higher risk of isolation and disruption [45]. In contrast, Pashazadeh Kan et al. [5] reported that each additional year of age was associated with a 0.3% decrease in QoL score, with lower average scores among adults over 60. This review linked lower QoL among older adults to physical limitations, social isolation, and heightened vulnerability to COVID-19.

Among children and adolescents, age-related patterns were more mixed. Ahn’s review [36] suggested that older adolescents may experience lower QoL, particularly in psychological and school-related domains, though findings were not consistently disaggregated. Orban et al. [37] reported that some studies found sharper declines in QoL over time among older children and adolescents. However, Nobari et al. [38] found greater increases in the proportion of low HRQoL among younger adolescents (11–13) than older adolescents (14–17), suggesting that younger age groups may have experienced a steeper relative decline in perceived QoL during the pandemic. Overall, the evidence suggests that younger adults and adolescents were generally more vulnerable to declines in QoL during the pandemic, while older adults showed mixed outcomes, experiencing both heightened risks and, in some contexts, relatively stable or higher QoL compared to younger groups.

#### 3.8.2. Gender

In the general population, Nshimirimana et al. [25] reported that women consistently had lower scores on both physical and mental health-related QoL measures (e.g., SF-36) compared to men. This gender disparity was observed across diverse countries and populations and was interpreted as reflecting a greater psychological burden borne by women during the pandemic. Similarly, Pashazadeh Kan et al. [5] found a statistically significant difference in pooled QoL scores, with men averaging 69.09 and women 61.08, indicating that women had notably poorer QoL during the pandemic period.

Among children and adolescents, several reviews reported similar findings. Ahn’s review [36] found that girls reported significantly lower HRQoL scores across physical, psychological, and school-related domains in multiple studies, including those using KIDSCREEN [56] and PedsQL [42,50]. Orban et al. [37] also reported evidence that girls had lower initial HRQoL and experienced steeper declines over time than boys, based on longitudinal data such as the COPSY study [51,57]. In contrast, Nobari et al. [38] found no consistent gender differences across the six included studies, although some studies reported slightly higher emotional symptoms in girls and better social functioning in boys.

Other reviews, covering working-age adults and older adults either did not report gender-specific findings [14] or mentioned them only anecdotally without supporting data [39]. Overall, the evidence suggests a clear pattern of lower QoL among females during the pandemic, particularly among adolescents and in the context of mental and emotional well-being. However, a few reviews found mixed or non-significant gender differences, highlighting the need to interpret these findings in light of population group and measurement tools.

#### 3.8.3. Socioeconomic Status

Three reviews identified socioeconomic status as a key contributor to reduced QoL during the COVID-19 pandemic, particularly among lower-income groups and populations in low- and middle-income countries [5,25,35]. El Keshky et al. [35] described how job loss, income insecurity, and rising poverty levels undermined psychological resilience and diminished living standards, with clear implications for perceived QoL. The review emphasised that these effects were most acute in economically fragile settings, where financial strain compounded the broader challenges of lockdown and health system disruption. While framed conceptually through the lens of “psychological sustainability,” the review treated this as closely aligned with QoL.

Nshimirimana et al. [25] found that lower socioeconomic status, defined through income, education, and employment, was consistently associated with lower HRQoL scores. Although economic stress was not the primary focus of the review, several included studies reported that individuals facing financial hardship or job insecurity experienced poorer outcomes across physical and mental QoL domains, suggesting a clear link between economic vulnerability and reduced perceived well-being. Pashazadeh Kan et al. [5] observed that regions with greater economic vulnerability, such as SEARO and AFR, reported the lowest average QoL scores during the pandemic. Subgroup analyses showed that patients with chronic conditions, and those with less employment stability also reported lower QoL. Although income was not an explicit grouping variable, the review interpreted its findings as reflecting broader structural inequalities that shaped QoL outcomes globally.

Evidence on education level as a determinant of QoL during the COVID-19 pandemic was limited as most reviews did not synthesise evidence on education level in relation to QoL outcomes. Only the review by Nshimirimana et al. [25] reported that higher education was associated with better HRQoL, though findings were not consistently disaggregated.

#### 3.8.4. Marital Status

Marital status was largely unexamined in the reviews included. Nshimirimana et al. [25] noted that it was not systematically analysed across the primary studies they reviewed, and no other included reviews reported any findings on QoL differences by marital status.

## 4. Discussion

### 4.1. Summary of Main Findings

This umbrella review synthesised evidence from nine reviews encompassing 272 primary studies, demonstrating that the COVID-19 pandemic had a substantial impact on multiple domains of QoL, including psychological, physical, social, and environmental aspects. While public health measures such as lockdowns and social distancing were essential for limiting viral transmission, they also disrupted everyday routines, social relationships, and access to services. These disruptions contributed to increased loneliness and emotional strain, which in turn negatively affected subjective perceptions of vitality, daily functioning, and life satisfaction.

Reviews of general population studies identified that reduced access to healthcare, lower levels of physical activity, and financial stressors such as job loss were consistently associated with declines in both physical and perceived overall QoL [25]. Similarly, restrictions on movement and the closure of recreational and public spaces contributed to reduced opportunities for leisure and social interaction, leading to increased feelings of isolation and reduced life satisfaction [7,43,53]. Environmental and socioeconomic conditions also played a significant role in shaping QoL outcomes during the pandemic. Individuals living in overcrowded housing or without access to green space reported poorer subjective QoL, suggesting that constrained physical environments may amplify psychological strain and limit coping resources [7,55].

Across the life course, reviews consistently reported declines in QoL during the pandemic, though patterns varied by age group. Among children and adolescents, reviews highlighted significant reductions in emotional and social functioning, with factors such as school closures, disrupted routines, and diminished peer interaction contributing to lower psychological and physical QoL. Longitudinal studies indicated that these impacts often persisted beyond the initial lockdown periods. Working-age adults were particularly affected by heightened psychological distress and reduced physical functioning, often linked to employment disruption, health anxiety, and decreased access to routine care and physical activity. Across studies, both emotional well-being and vitality were commonly reported as diminished.

Among older adults, restrictions on mobility and limited access to healthcare services were closely linked to reduced daily functioning and increased loneliness, reinforcing a cyclical relationship between social isolation, emotional well-being, and physical health [14,43]. For example, studies from Austria reported that heightened loneliness during lockdowns was strongly associated with reduced emotional well-being and life satisfaction, which in turn negatively impacted perceived vitality and overall QoL [58]. Other evidence suggests that QoL among older adults was influenced by contextual factors such as marital status, living arrangements, and access to social support [59,60]. These findings indicate that declines in QoL during the COVID-19 pandemic were shaped by interrelated stressors across psychological, physical, and environmental domains, often compounding each other to reinforce disadvantage.

### 4.2. Findings in Relation to Other Umbrella Reviews of Public Health Impacts

Several recent umbrella reviews have similarly examined the broader health impacts of the COVID-19 pandemic, offering useful context for interpreting our findings. Bower et al. [61], in a synthesis of 338 systematic reviews, reported elevated rates of anxiety, depression, and psychological distress across diverse populations, with particularly high burden among healthcare workers, young people, and individuals directly affected by COVID-19. Duan et al. [15] focused specifically on children and adolescents, highlighting strong associations between the pandemic and adverse mental health outcomes. However, both reviews noted that the methodological quality of much of the available evidence was low, and that QoL was often underexamined compared to mental health.

Findings from other umbrella reviews further illustrate the breadth of pandemic-related health impacts, particularly on mental health. Witteveen et al. [62] reported small but consistent increases in depression and anxiety symptoms in the general population during the early pandemic, especially during periods of strict social restrictions, with stronger effects observed among women and younger adults. Similarly, Mohseni et al. [63] identified higher prevalence of mental health symptoms such as stress and PTSD in certain populations, but also noted variability by region and study quality. Umbrella reviews by Dragioti et al. [64] and Fernandez et al. [33] focused on healthcare workers globally, who reported high rates of anxiety, depression, and burnout. Across these reviews, methodological limitations and the lack of longitudinal data constrained efforts to draw firm conclusions about long-term effects, but collectively the findings illustrate the wide-ranging and inequitable impacts of the pandemic on mental health and QoL. These results point to the importance of strengthening surveillance systems, prioritising vulnerable populations in crisis response planning, and standardising QoL metrics to support cross-context comparison and timely policy responses in future public health emergencies.

### 4.3. Methodological Limitations and Evidence Gaps

Although a substantial volume of research has examined QoL during the COVID-19 pandemic, several methodological limitations constrain the ability to draw generalisable conclusions. Notably, relatively few reviews focused specifically on the general population. Instead, much of the literature concentrated on particular subgroups, such as individuals with long COVID, children and adolescents, or people with pre-existing health conditions. This limits the synthesis of findings that apply across the full population. Furthermore, as with all umbrella reviews, the synthesis was limited by the level of detail reported in the included reviews, which often summarised findings across diverse primary studies without consistently reporting specific outcome data or subgroup analyses. This umbrella review protocol was not registered prospectively in PROSPERO, which may limit transparency.

Another key limitation is the heterogeneity in how QoL was defined and measured across studies. Reviews included a wide range of instruments, such as the SF-36, EQ-5D, WHOQOL-BREF, and KIDSCREEN, each capturing different dimensions and with varying emphasis on physical, psychological, and social domains. The lack of a standardised framework complicates cross-study comparisons and makes it difficult to assess the relative contribution of different factors to overall QoL.

Most included primary studies were cross-sectional in design, limiting the ability to understand changes over time or assess long-term impacts. Furthermore, most reviews included in this umbrella synthesis focused on data collected during the early and mid-stages of the COVID-19 pandemic, reflecting immediate impacts on QoL. However, some reviews, such as Orban et al. [37], included longitudinal studies that captured longer-term changes, suggesting that psychological and social disruptions to QoL may have persisted beyond initial lockdown periods.

Finally, the geographical distribution of studies was uneven, with some world regions, particularly low- and middle-income countries, underrepresented in the evidence base. This raises concerns about the global generalisability of findings and suggests that the full extent of the pandemic’s impact on QoL may not yet be fully captured. Furthermore, included reviews and primary studies were published in English, which may limit the generalisability of findings to non-English-speaking populations. Future evidence syntheses should consider including non-English sources to improve global representation and reduce language bias.

### 4.4. Future Research

Future research should prioritise the use of standardised and validated frameworks for measuring QoL to support comparability across studies and contexts. Greater use of longitudinal study designs is also needed to assess the trajectory of QoL over time and to understand both short- and long-term impacts of public health crises. There is a particular need for studies that explore protective factors associated with better QoL outcomes, such as individual resilience, health literacy, and supportive environments. These factors are often underexamined yet may play a crucial role in buffering the adverse effects of pandemic-related disruptions. Research should also aim to better capture the experiences of populations in low- and middle-income countries, where the impacts of the pandemic may differ in scope and severity due to differences in infrastructure, healthcare systems, and social safety nets.

While some reviews reported gender-based differences in QoL among children and adolescents, there was limited evidence disaggregating other sociodemographic factors such as socioeconomic status across different age groups. Future research should explore how these factors intersect with age to shape experiences of QoL during public health crises. Addressing these gaps will strengthen the evidence base, improve the synthesis of findings, and help inform targeted, equitable strategies to protect population well-being during future public health emergencies.

### 4.5. Public Health Implications

This review highlights how QoL during the pandemic varied substantially across population groups and contexts. Differences in policy responses, healthcare system capacity, and social protections shaped the extent to which individuals and communities were able to maintain QoL. Evidence suggests that countries with stronger governance, well-resourced health systems, and effective social protection mechanisms reported better QoL outcomes, while populations in settings with weaker infrastructure experienced more pronounced declines [5,35].

Across studies, QoL was consistently described as a multidimensional construct, encompassing physical, psychological, social, and environmental domains. This highlights the limitations of single-sector responses. For example, the availability of accessible outdoor environments supported physical activity and mitigated psychological distress [7,65]. Economic policies that protected employment or provided income support helped to reduce material hardship and related mental stress [66]. Digital inclusion initiatives enabled continued access to services and helped maintain social connectivity during periods of physical isolation [67]. These findings suggest that safeguarding QoL during public health emergencies requires coordinated, multisectoral approaches that integrate health, economic, social, and environmental strategies. Future preparedness efforts should prioritise policies that reduce inequality, build resilience, and invest in infrastructure capable of supporting population well-being under crisis conditions.

## 5. Conclusions

This umbrella review demonstrates that the COVID-19 pandemic was associated with a widespread and multidimensional decline in QoL across global populations. Psychological, physical, social, and environmental domains were all affected, with evidence of disproportionate impacts among women, younger individuals, those with lower socioeconomic status, and populations in settings with limited infrastructure or support systems.

The findings highlight the interrelated nature of QoL domains and the cumulative effects of pandemic-related stressors. Addressing these challenges requires holistic, cross-sector strategies that extend beyond health services alone. Building more resilient health systems and communities will depend on integrated approaches that support mental well-being, reduce economic hardship, promote digital and environmental equity, and prioritise those most at risk of decline in QoL during future public health emergencies.

## Figures and Tables

**Table 3:** Results of Quality Appraisal Using the JBI Critical Appraisal Checklist for Systematic Reviews and Research Syntheses.

First Author	El Keshky	Kasar	Melo-Oliveira	Mouratidis	Nae Ahn	Nobari	Nshimirimana	Orban	Pashazadeh Kan
Is the review question clearly and explicitly stated?	Yes	Yes	Yes	Yes	No	Yes	Yes	Yes	Yes
Were the inclusion criteria appropriate for the review question?	Yes	Yes	Yes	No	Yes	Yes	Yes	Yes	Yes
Was the search strategy appropriate?	No	Yes	Yes	Unclear	Yes	Yes	Yes	Yes	Yes
Were the sources and resources used to search for studies adequate?	Yes	Yes	Yes	Unclear	Yes	Yes	Yes	Yes	Yes
Were the criteria for appraising studies appropriate?	No	N/A	No	No	Yes	Yes	Yes	Yes	Yes
Was critical appraisal conducted by two or more reviewers independently?	N/A	N/A	Yes	Unclear	Unclear	Yes	Yes	Yes	Yes
Were there methods to minimize errors in data extraction?	Unclear	Yes	Yes	Unclear	Unclear	Yes	Yes	Yes	Yes
Were the methods used to combine studies appropriate?	Unclear	N/A	Unclear	Unclear	Yes	Yes	Yes	Yes	Yes
Was the likelihood of publication bias assessed?	N/A	N/A	No	N/A	N/A	N/A	Yes	N/A	Yes
Were recommendations for policy/practice supported by reported data?	Yes	Yes	Unclear	Unclear	Unclear	Unclear	Unclear	Unclear	Unclear
Were the specific directives for new research appropriate?	No	Unclear	Yes	No	Yes	Yes	Yes	Unclear	Unclear
Total Score	44%	86%	64%	10%	60%	90%	91%	80%	82%
Overall quality appraisal	Medium	High	Medium	Low	Medium	High	High	High	High

## Data Availability

No new data were created or analyzed in this study. Data sharing is not applicable to this article.

## Supplementary Materials

The following supporting information can be downloaded at: https://www.mdpi.com/article/10.3390/jcm14238348/s1, Table S1: PRISMA checklist; Table S2: PRISMA Abstract checklist; Table S3: the PRIOR Checklist for Overviews of Reviews of Healthcare Interventions.

## Abbreviations

The following abbreviations are used in this manuscript:

AFRO	African Region (WHO)
AMRO	Region of the Americas (WHO)
CCA	Corrected Covered Area
COVID-19	Coronavirus Disease 2019
EPHPP	Effective Public Health Practice Project
EQ-5D	EuroQol Five-Dimension Scale
EQLS	European Quality of Life Survey
EURO	European Region (WHO)
GHQ-12	General Health Questionnaire-12
HRQoL	Health-Related Quality of Life
ICT	Information and Communication Technology
IES-15	Impact of Event Scale-15
JBI	Joanna Briggs Institute
KIDSCREEN	KIDSCREEN Health-Related Quality of Life Questionnaire
PedsQL	Pediatric Quality of Life Inventory
PHQ-9	Patient Health Questionnaire-9
PSQI	Pittsburgh Sleep Quality Index
QoL	Quality of Life
SEARO	South-East Asia Region (WHO)
SF-8	Short Form Health Survey-8
SF-12	Short Form Health Survey-12
SF-36	Short Form Health Survey-36
SRQ	Self-Reporting Questionnaire
WPRO	Western Pacific Region (WHO)
WHO	World Health Organization
WHOQOL-BREF	World Health Organization Quality of Life—Brief
