# Supplementary material for: An Umbrella Review of Quality of Life Among the General Population During the COVID-19 Pandemic
Source: J Clin Med. 2025 Nov 24;14(23):8348. doi: 10.3390/jcm14238348 (PMC12693030; doi:10.3390/jcm14238348)
Supplement: Supplementary file 1 [file jcm-14-08348-s001.zip › Table S1-PRISMA-2020-checklist.pdf]

PRISMA

PRISMA 2020 Checklist

Section and Topic	Item #	Checklist item	Location where item is reported
**TITLE**			
Title	1	**An Umbrella Review of Quality of Life Among the General Population During the COVID-19 Pandemic**	Lines 2-3
**ABSTRACT**			
Abstract	2	1. Objectives<br>2. Methods<br>3. Results<br>4. Conclusions	10-12<br>12-17<br>17-25<br>25-28
**INTRODUCTION**			
Rationale	3	Existing knowledge	53-88
Objectives	4	Statement of aims and objectives.	89-96, 99-104
**METHODS**			
Eligibility criteria	5	Inclusion and exclusion criteria for the review and how studies were grouped for the syntheses.	112-151
Information sources	6	Databases searched	162-166
Search strategy	7	Search strategies for all databases, registers and websites, including any filters and limits used.	166-177
Selection process	8	Study selection	179-191
Data collection process	9	Data extraction	213-214
Data items	10a	Outcome	219-21
	10b	Other variables	215-218
Study risk of bias assessment	11	Quality appraisal	196-211
Effect measures	12	N/A	
Synthesis methods	13a	N/A	
	13b	N/A	
	13c	N/A	
	13d	Data synthesis	223-232
	13e	Describe any methods used to explore possible causes of heterogeneity among study results (e.g. subgroup analysis, meta-regression).	
	13f	N/A	
Reporting bias assessment	14	N/A	
Certainty assessment	15	N/A	
**RESULTS**			

PRISMA

PRISMA 2020 Checklist

Section and Topic	Item #	Checklist item	Location where item is reported
Study selection	16a	Flow diagram	Fig 1
	16b	N/A	
Study characteristics	17	Cite each included study and present its characteristics.	Table 1a/b
Risk of bias in studies	18	Quality appraisal	Table 2
Results of individual studies	19	N/a	
Results of syntheses	20a	For each synthesis, briefly summarise the characteristics and risk of bias among contributing studies.	Tables 1a/b, 2
	20b	N/A	
	20c	N/A	
	20d	N/A	
Reporting biases	21	N/A	
Certainty of evidence	22	N/A	
**DISCUSSION**			
Discussion	23a	General interpretation of the results in the context of other evidence.	597-657
	23b	Discuss any limitations of the evidence included in the review.	670-690
	23c	Limitations	659-669
	23d	Implications of the results for practice, policy, and future research.	692-729
**OTHER INFORMATION**			
Registration and protocol	24a	N/A	
	24b	N/A	
	24c	N/A	
Support	25	N/A	
Competing interests	26	None	
Availability of data, code and other materials	27	On request	

From: Page MJ, McKenzie JE, Bossuyt PM, Boutron I, Hoffmann TC, Mulrow CD, et al. The PRISMA 2020 statement: an updated guideline for reporting systematic reviews. BMJ 2021;372:n71. doi: 10.1136/bmj.n71.
This work is licensed under CC BY 4.0. To view a copy of this license, visit https://creativecommons.org/licenses/by/4.0/
